# Supplementary material for: Comirnaty-Elicited and Convalescent Sera Recognize Different Spike Epitopes
Source: Vaccines (Basel). 2021 Dec 1;9(12):1419. doi: 10.3390/vaccines9121419 (PMC8708883; doi:10.3390/vaccines9121419)
Supplement: Supplementary file 1 [file vaccines-09-01419-s001.zip › vaccines-1447500-supplementary.pdf]

vaccines

MDPI

Supplementary:

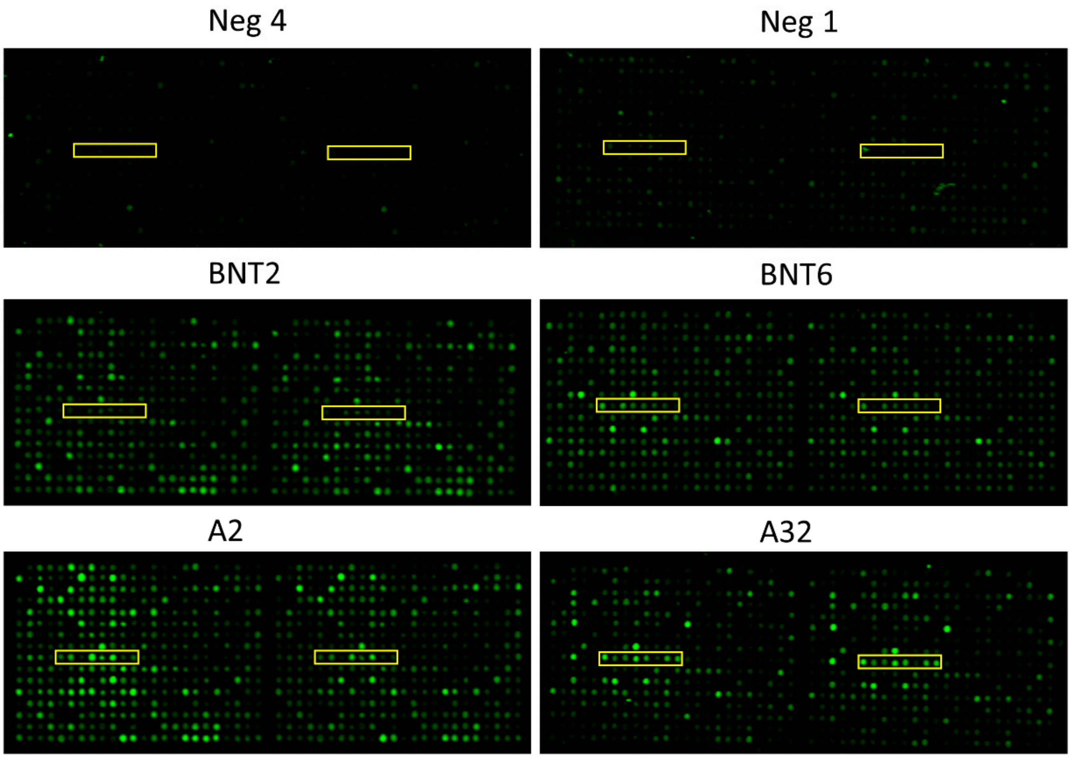

Figure S1. Representative peptide arrays. The spots representing the central helix are marked in yellow. Top: CRTL samples, middle: vaccinated samples, down: convalescent sera.

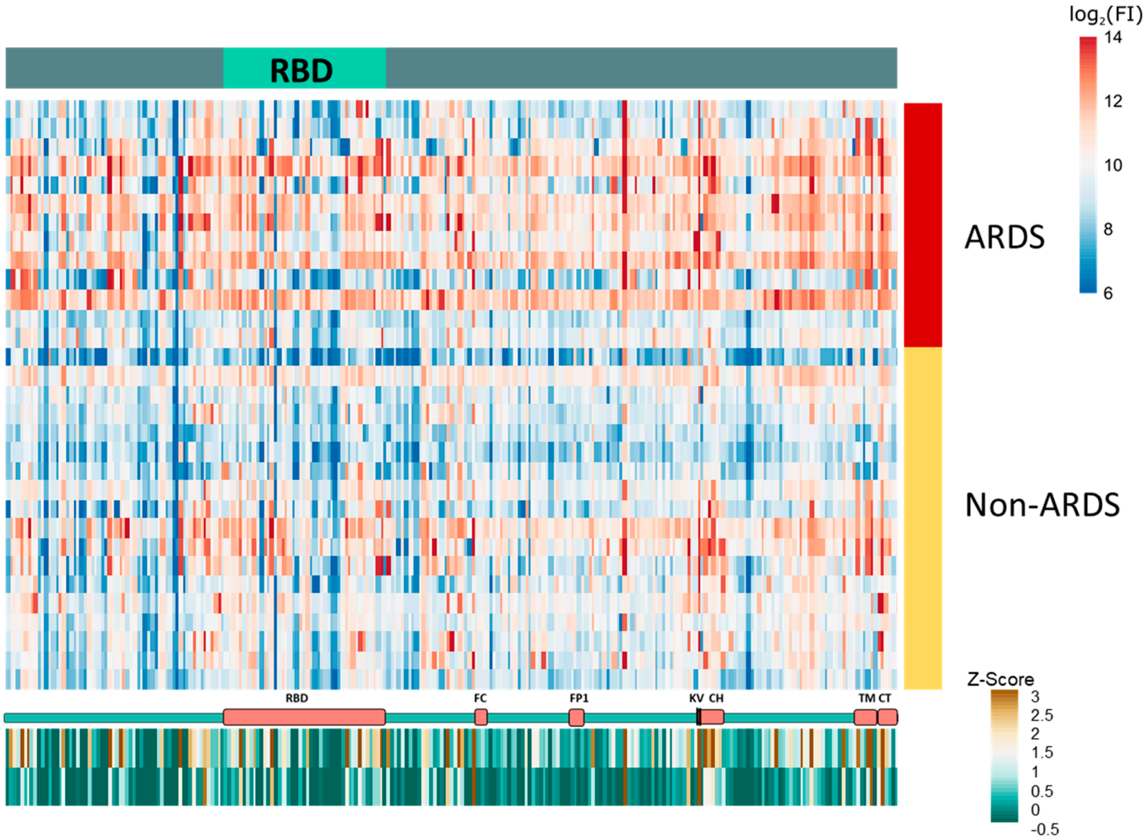

Figure S2. Mapping of linear S protein epitopes (non-ARDS vs. ARDS). A, Heatmap of IgG antibody immune response of 31 sera from COVID-19 convalescent patients (non-ARDS (n=18) and ARDS (n=13)) and 4 healthy donors. FI: fluorescence intensity. B-C, Schematic structure of the S protein and condensed heatmap of all 56 sera grouped in vaccinated and convalescent patients. RBD: Receptor binding domain, FC: Furin cleavage site, FP: Fusion peptide, KV: Amino acid positions K986 and V987, which are mutated to proline in the pre-fusion stabilized S protein, CH: Central helix, TM: Transmembrane domain, CT: cytoplasmic tail.

Table S1. Exclusively in ARDS patients identified linear S protein epitopes. Red: Z-score >=1.5.

Location-Peptide Nr.	Z-Score ARDS	Z-Score non-ARDS	Peptide
S-2	2.5	−0.1	VLLPLVSSQCVNLTT
S-3	1.7	0.2	VSSQCVNLTTRTQLP
S-5	3.7	0.7	RTQLPPAYTNSFTRG
S-7	3.4	1.3	SFTRGVYYPDKVFRS
S-17	2.5	1.0	NPVLPFNDGVYFAST
S-24	2.1	0.4	SLLIVNNATNVVIKV
S-30	6.6	1.0	HKNNKSWMESEFRVY
S-31	1.5	0.2	SWMESEFRVYSSANN
S-33	4.5	0.5	SSANNCTFEYVSQPF
S-34	2.2	0.6	CTFEYVSQPFLMDLE
S-45	1.9	0.4	SALEPLVDLPIGINI
S-50	5.3	1.4	RSYLTPGDSSSGWTA
S-53	2.3	0.3	GAAAYYVGYLQPRTF
S-54	1.8	1.4	YVGYLQPRTFLLKYN

S-58	1.9	0.7	TDAVDCALDPLSETK
S-61	1.9	1.0	CTLKSFTVEKGIYQT
RBD-65	1.5	0.1	QPTESIVRFPNITNL
RBD-69	1.7	0.5	VFNATRFASVYAWNR
RBD-71	2.4	1.2	YAWNRKRISNCVADY
RBD-74	1.6	0.6	SVLYNSASFSTFKCY
RBD-79	1.6	0.9	CFTNVYADSFVIRGD
RBD-80	1.7	0.7	YADSFVIRGDEVRQI
RBD-86	2.3	0.6	PDDFTGCVIAWNSNN
RBD-97	3.1	1.3	NGVEGFNCYFPLQSY
RBD-101	2.9	1.2	NGVGYQPYRVVVLSF
RBD-103	1.9	0.3	VVLSFELLHAPATVC
RBD-105	2.0	0.4	PATVCGPKKSTNLVK
RBD-106	1.9	0.4	GPKKSTNLVKNKCVN
S-109	2.6	0.7	FNFNGLTGTGVLTES
S-119	1.7	1.4	SFGGVSVITPGTNTS
S-120	1.9	0.8	SVITPGTNTSNQVAV
S-129	3.3	1.3	NVFQTRAGCLIGAEH
S-130	2.3	0.5	RAGCLIGAEHVNNSY
S-148	1.6	0.6	VDCTMYICGDSTECS
S-167	2.6	0.6	AGFIKQYGDCLGDIA
S-184	2.1	0.1	LYENQKLIANQFNSA
S-199	3.1	1.3	VQIDRLITGRLQSLQ
S-200	2.4	1.2	LITGRLQSLQTYVTQ
S-202	1.8	0.3	TYVTQQLIRAAEIRA
S-203	2.0	0.2	QLIRAAEIRASANLA
S-218	1.6	0.5	KAHFPREGVFVSNGT
S-219	2.9	0.0	REGVFVSNGTHWFVT
S-223	1.8	0.2	EPQIITTDNTFVSGN
S-226	2.9	1.0	CDVVIGIVNNTVYDP
S-227	1.5	0.1	GIVNNTVYDPLQPEL
S-231	1.5	0.1	ELDKYFKNHTSPDVD
TM-242	2.8	0.9	YEQYIKWPWYIWLGF
TM-245	3.4	0.7	IAGLIAIVMVTIMLC
TM-248	1.9	1.3	CMTSCCSCLKGCCSC
CT-250	2.0	0.3	GCCSCGSCCKFDEDD
